# Pilot Study of the Shade Matching of Biomimetic Composite Resins in Posterior Dental Restorations: Randomised Clinical Trial

**DOI:** 10.3390/ma18122800

**Published:** 2025-06-14

**Authors:** Cristina Rico-Romano, Dina Aslimani Amar, Valentin Ducept, Rosa M. Vilariño-Rodríguez, Pablo Garrido-Martínez, Vanessa Gutierrez-Vargas, Jesús Mena-Álvarez

**Affiliations:** Faculty of Dentistry, Alfonso X El Sabio University, 28691 Madrid, Spain; cromaric@uax.es (C.R.-R.); dasliama@myuax.com (D.A.A.); valducept@outlook.fr (V.D.); rvilarod@uax.es (R.M.V.-R.); pgarrido@uax.es (P.G.-M.); vgutivar@uax.es (V.G.-V.)

**Keywords:** color, EasyShade, PrimeScan, shade matching, spectrophotometer

## Abstract

Cosmetic restorative dentistry focuses on restoring teeth affected by caries or trauma using materials that mimic natural teeth in shape, texture, and color. Composite resins, particularly nanofilled composites, are widely used due to their superior mechanical and aesthetic properties. Accurate tooth color selection is crucial, and methods include visual (shade guides) and instrumental (spectrophotometers, colorimeters, and intraoral scanners). Newer biomimetic composites, such as Admira Fusion 5 and Clearfil Majesty ES-2 Universal, simplify shade selection through advanced optical technologies. A randomized clinical study involving 30 patients compared the color-matching accuracy of two biomimetic composite resins: Admira Fusion 5 (Voco) and Clearfil Majesty ES-2 Universal (Kuraray). The study utilized the Vita Easyshade spectrophotometer and the Primescan intraoral scanner. Patients were treated following standardized protocols, and shade accuracy was evaluated pre- and post-restoration using Cohen’s Kappa index. Admira Fusion 5 showed higher shade-matching accuracy, with good agreement between pre- and post-restoration measurements using both instruments. Clearfil Majesty ES-2 Universal demonstrated lower reproducibility in shade matching, particularly in posterior teeth, with lower agreement in pre- and post-tests. Instrumentation Comparison: Primescan showed slightly better performance than Easyshade, but both provided comparable results. In conclusions, universal composites may not always achieve optimal shade matching in posterior teeth. Layered composites provide better color adaptability. While digital instruments enhance shade accuracy, combining them with visual methods yields the best clinical outcomes. Further research with larger sample sizes is needed to improve shade-matching techniques in aesthetic restorative dentistry.

## 1. Introduction

Aesthetic restorative dentistry is responsible for the total or partial restoration of a tooth affected by caries or trauma using biomaterials that mimic the natural appearance of teeth, thereby improving the smile in terms of shape, texture, and color. Therefore, the appropriate selection of the most suitable material as well as the tooth color is very important when carrying out a correct treatment.

Composite resins are the materials most commonly used in aesthetic restorative dentistry, both in the anterior sector, due to their good polishability and properties (translucency/opacity, fluorescence, …), and in the posterior sector, mainly due to their mechanical resistance. For this reason, improvements and modifications to these materials are constantly being made in order to achieve the simplest possible aesthetic restoration [1].

Currently, nanofilled composite resins are the most successful resins when direct aesthetic restoration is indicated. They are characterized by the reduction in size of the filler particles to nanometric dimensions (less than 10 nm (0.01 µm)). This has exponentially improved mechanical and aesthetic properties, such as abrasion resistance, surface texture, translucency, and polishability. This makes them suitable for both the anterior and posterior sectors. These resins are modified to obtain the color, translucency, and opacity of natural teeth, thus achieving a much more aesthetic material for our restorations. It is also essential to select the right tooth shade to achieve this objective [2].

We have two methods for assessing tooth color: visual (subjective) and instrumental (objective) methods. The visual method of color estimation has been one of the most widely used for a long time and is used to compare the color of the tooth with samples from an artificial shade guide [3]. The most commonly used shade guides are Vita Guide Classic, which, among its disadvantages, does not represent all possible colors of natural teeth; and ToothGuide 3D Master, which, through sorting by value, saturation, and hue, in this order, is intended to facilitate the work of the human eye according to its capabilities, in order to obtain the best results [4].

Recently, a range of electronic instruments designed to facilitate and objectify this process have appeared on the market: colorimeters [5], spectrophotometers [6], and digital cameras with imaging systems [7]. Digital spectrophotometers are the most accurate instruments in dentistry for recording color. Their major advantage is their objectivity, which provides an accurate assessment, saving time and visits [8]. The spectrophotometer is 33% more accurate and precise than the human eye. However, its high cost and complicated operation have hindered its use in the dental clinic [9].

At the same time, manufacturers offer state-of-the-art universal restorative materials, including biomimetic composites, such as Admira Fusion 5 (Voco) and Clearfil Majesty ES-2 Universal (Kuraray), among others. These composites offer a simplified shade system by means of shade matching. This allows adaptation to the natural shade gradient, as well as easier and more efficient shade selection [10]. Thanks to the simplified shade system, it is possible to cover all 16 shades of the VITA classical scale with only five cluster shades. No overlapping of several colors is necessary.

Admira Fusion 5 features a new, patented nano-hybrid ORMOCER resin matrix, which, through its precise adaptation to the size and optical properties of the nano-hybrid particles, achieves optimized light scattering. The result is a defined and enhanced chameleon effect, which allows the restorative material to perfectly match the natural tooth shade.

Silicon oxide is the chemical basis of both the filler and the resin matrix. This “Pure Silicate Technology” also achieves several important advantages: the lowest level of polymerization shrinkage (1.25 vol. %), excellent biocompatibility, and very low shrinkage stress compared to all conventional restorative composites, which is associated with an optimal marginal seal. In addition, thanks to its low water absorption (9.1 g/mm^3^), it achieves high shade stability, guaranteeing very aesthetic results in the long term. Due to its soft, non-sticky consistency, it adapts very well to cavity walls and is easy to shape. It has a quick and easy high-gloss polish, which, in combination with its excellent mechanical properties and high flexibility, makes it an excellent choice for long-term aesthetic restorations in anterior and posterior teeth.

Clearfil Majesty differs from the previous materials in its technology and number of shades; it has a single universal shade for posterior teeth, regardless of the underlying base shade [11]. Thanks to Kuraray Noritake Dental’s light scattering technology, the restorations distort light in a similar way to dental tissue. The composite integrates easily and smoothly with the surrounding tooth structure and even dark spots on the cavity floor become invisible under the material, which absorbs, scatters, and diffuses light correctly. In the aesthetically more demanding anterior region, there are two shades to choose from.

The objectives of this study were twofold: The first was to compare the measurement of dental color using a digital device (Primescan; Dentsply Sirona, York, PA, USA), a spectrophotometer (Easyshade Vita: Vita Zahnfabrik, Bad Sackingen, Germany), and the Vita Classic shade guide. The second was to evaluate the final shade of posterior composite restorations made using the layering technique with Kuraray Clearfil Majesty ES-2 Universal (Kuraray Noritake Dental, Tokyo, Japan) and Admira Fusion 5 (Voco GmbH, Cuxhaven, Germany) with Kuraray Clearfil Majesty ES-2 Universal. The entire study was conducted by dental students.

## 2. Materials and Methods

This was a randomized clinical study with 30 patients, 15 of whom were rehabilitated with Clearfil Majesty TM ES-2 (universal shade) biomimetic composite resin from Kuraray, Japan and 15 with Admira Fusion 5 from Voco, Germany. The Vita Easyshade spectrophotometer (Vita Zahnfabrik, Bad Sackingen, Germany) and the Primescan intraoral scanner (Dentsply Sirona) were used for shade evaluation and comparison.

The study was carried out at the Undergraduate Dental Clinic of the Faculty of Dentistry of the Alfonso X el Sabio University (UAX) and lasted 6 months (October–May 2024). The 30 patients that were included met the following inclusion criteria: over 18 years of age, presence of class 1 and 2 caries in posterior teeth, good periodontal status, no previous treatment on the tooth to be restored, said teeth could be fitted with absolute isolation, and sufficient mouth opening for access with the Primescan scanner and Vita Easyshade spectrophotometer. As exclusion criteria, all patients who, even though they met the inclusion criteria, required root canal treatment or pulp capping were excluded.

To minimize slants and ensure that the differences observed in the results are truly attributable to the intervention or to the variable that has being studied (type of resin and type of shade taking), we performed a single-blind randomization using the Epidat 4.2 program of Epidemiology Service of the General Directorate of Public Health of the Ministry of Health (Galicia/Spain). In this way, we were able to divide the cases into two groups: (a) patients treated with Admira Fusion 5 19, 20, 27, 8, 29, 9, 12, 21, 28, 15, 5, 3, 14, 24, or 11, and (b) patients treated with Clearfil Majesty ES-2 1, 2, 4, 6, 7, 10, 13, 16, 17, 18, 22, 23, 25, 26, 27, or 30.

The study was approved by the UAX Ethics and Research Committee (Annex 1). All participants voluntarily accepted inclusion in the study and signed the Information Sheet and Informed Consent, following the recommendations of the Helsinki convention.

The work protocol was carried out by a single operator and the steps were as follows:

1. Bestdent nylon prophylaxis brush on the tooth to be treated.
2. Application of local anesthesia in cases where the depth of the caries required it.
3. Installation of absolute insulation with Proclinic rubber dam.
4. Caries cleaning and cavity creation.
5. Removal of rubber dam for color determination and wait 2 min before the test.
6. Color measurement with the Vita Easyshade spectrophotometer and the Primescan intraoral scanner at the center of the clinical crown from occlusal, repeating the same measurement three times.
7. Choice of composite resin shade in cases where the filling was carried out with Admira Fusion 5 composite from Voco, Germany. Installation of absolute insulation with Proclinic rubber dam and layering of the material in 2 mm thicknesses; final polishing with milling cutter and polishing rubber from Edenta.
8. Removal of rubber dam and final color measurement with the Vita Easyshade spectrophotometer and the Primescan intraoral scanner at the occlusal center of the clinical crown, repeating the same measurement three times.
9. For the descriptive statistical study, mean, standard deviation, and maximum and minimum values were calculated. For the degree of concordance between the pre- and post-tests, Cohen’s Kappa index was used and based on Landis and Koch’s interpretation of the results.

The aim of this study was to determine the degree of similarity and equality between the pre- and post-color measurements of the 15 color samples taken, both descriptively and statistically. It also aimed to understand the composite performance between the pre- and post-color measurements with a broader sample of 30 cases. Three pre- and post-color measurements were taken for each machine. On the Prime machine, the color measurements were the same in all three samples, so it remained as a single value. Descriptively, the percentage of similarity between the pre- and post-color measurements for each of the samples was calculated for each of the study machines. Statistically, Cohen’s Kappa was used to study the degree of agreement or concordance between the pre- and post-color measurements. The interpretation of the statistic given by Landis and Koch is as follows: Values above 0.8 indicate excellent agreement; values between 0.6 and 0.8 indicate good agreement; and finally, values below 0.4 indicate poor agreement. In the analysis, significant levels must be found to reject the null hypothesis that the agreement is greater than expected by chance.

## 3. Results

### 3.1. Analysis of Machine Performance with Admira Fusion

For the Easyshade machine, at the percentage level, the highest coincidence was found in the pre- and post-color shots in shots 2 and 3. Likewise, the Kappa agreement was observed as good (values between 0.6 and 0.8) (Table 1).

For the Prime machine, it can be observed that the color scores match 66.7% of the time (Table 2). Similarly, the null hypothesis of zero agreement can be rejected, and the agreement index was observed to be good (k = 0.626).

### 3.2. Analysis of the Performance of Machines with Clearfil Majesty TMES-2

With the Clearfil Majesty TM ES-2 resin, a good concordance index was not found when comparing pre- and post-test results with the Easyshade and Primescan machines. (Table 3 and Table 4).

### 3.3. Performance Analysis of Composite Resins

As can be seen in Table 5, the percentage of coincidence in the three color shots collected pre- and post-test was 36.7% of the cases (33 hits out of 90), with a higher percentage of coincidence found in the third shot (46.7%). These results are statistically supported by Cohen’s Kappa index.

According to the analysis of the different determination of the two composites, it can see that there was a high difference and the best-performing composite was Admira Fusion 5 from Voco, with a lower percentage than Clearfil Majesty TM ES-2 from Kuraray.

As can be seen in Table 6, the percentage of agreement between the three color samples collected pre- and post-tests was 42.2% (38 correct answers out of 90), with the third sample showing the highest percentage of agreement, reaching 50.0%. These results were statistically supported by Cohen’s Kappa coefficient, which shows statistically significant results for all samples collected, with indices above 0.4 in the second and third samples, indicating good agreement between the pre- and post-test samples.

## 4. Discussion

The primary aim of this study was to evaluate the shade-matching ability of two biomimetic composite resins in posterior dental restorations and to compare different shade selection methods. The results obtained, although preliminary due to the limited sample size (n = 15), suggest that the Kuraray Clearfil Majesty ES-2 Universal composite failed to accurately reproduce the tooth shade in a significant number of cases, both with the Easyshade spectrophotometer and the Primescan intraoral scanner. An exact match was observed in four out of 15 cases with Easyshade and in five out of 15 cases with Primescan, indicating limited reproducibility in shade selection. This can be attributed to the characteristics of the submicron filler particles (nano- and microhybrids) that provide translucency, brilliance, and naturalness, to the high-tech matrix that maximizes light transmission and color stability, and to its strong chameleon effect that is specifically formulated to adapt to the color of the natural tooth.

These findings align with previous studies assessing the color matching performance of monochromatic universal composites. For instance, Iyer et al. reported similar outcomes in a study comparing shade reproduction between universal and layered composites, noting that Tokuyama’s Omnichroma showed lower color-matching ability compared to traditional composites [10]. Similarly, Abreu et al. concluded that while universal composites perform well on lighter teeth, they struggle to match darker shades, particularly in posterior regions [11]. This limitation is partially attributed to the structural composition of universal resins, which lack pigments and rely solely on optical properties to blend with surrounding tooth structure.

In discussing the method, we must emphasize that shade sampling must be performed before complete isolation, since isolation causes dehydration within a few seconds (<2 min) and affects the shade sampling process. Therefore, in our process, a rubber dam was installed to create the cavity and then removed to allow color sampling [12,13,14].

The difficulty in reproducing an accurate shade in posterior restorations can be explained by several factors inherent to tooth morphology and light interaction. As noted by Tabatabaian et al., the curved and angled surfaces typical of posterior teeth create light reflections that hinder accurate shade determination, an issue affecting both spectrophotometers and intraoral scanners [6]. Severe discolorations, such as those caused by advanced caries or enamel defects, further compromise the chromatic adaptation of universal composites. In such cases, the composite often appears too light compared to the original tooth, resulting in suboptimal aesthetic outcomes.

As for the performance of Admira Fusion 5, another composite evaluated, it was superior to Kuraray, showing better results in terms of shade matching. This can be attributed to the characteristics of the submicron filler particles (nano- and microhybrids) that provide translucency, brilliance, and naturalness, to the high-tech matrix that maximizes light transmission and color stability, and to its strong chameleon effect that is specifically formulated to adapt to the color of the natural tooth. These results support the notion that layered composites, which include several shades, offer greater flexibility and precision by combining shades that are better adapted to the chromatic variations of the tooth. Studies by Browning et al. and Ryan et al. highlight the limitations of universal resins when compared to standardized shade guides, such as the Vita Classical guide, indicating that results with these materials often fall short of established standards [15,16].

Another relevant variable is the composition of the composite resins. The proportion of Bis-GMA, which is present in a higher proportion in classical layered composites, influences the translucency and light reflectance of the material. A lower proportion or absence of Bis-GMA in some universal composites, such as Omnichroma and Kuraray, may contribute to their reduced ability to mimic natural tooth structure. This observation aligns with studies such as that of Pereira Sánchez et al., who concluded that universal composites have limitations in matching color under complex clinical conditions [3].

Regarding shade selection methods, the present study found that both the Easyshade spectrophotometer and the Primescan scanner yielded comparable results but with technical differences: Easyshade prioritizes color accuracy, but its inter-device agreement is limited (33.3% in VITA classification), while Primescan optimizes geometric accuracy, especially at the preparation margins, with Primescan performing slightly better in an additional case. However, several studies suggest that spectrophotometers remain the most accurate choice for color selection. Ebeid et al. compared the accuracy of several intraoral scanners with the Vita Easyshade spectrophotometer and concluded that, although the scanners offer acceptable accuracy, the spectrophotometer remains more reliable for color pick-up [8]. These findings are consistent with those of Alsaleh et al., who demonstrated that digital instrumental methods such as spectrophotometry provide greater accuracy than visual assessments, which are susceptible to subjective factors like eye fatigue or lighting conditions [17].

Although digital devices, such as the Easyshade spectrophotometer and the Primescan intraoral scanner, have shown advantages in terms of accuracy, it is important to note that, according to Witkowski et al., color acquisition by these methods can be affected by external factors, such as lighting conditions and the angle of color acquisition. This aspect is relevant to improve reproducibility and accuracy in the clinical setting, but as several authors point out, the combination of digital and visual methods remains the most reliable strategy [18].

Despite technological advances, shade acquisition remains a challenge in restorative dentistry, especially in posterior teeth. The complex interaction of factors such as translucency, the angle of incidence of light, and the reflective properties of the tooth, described by Karamouzos et al., directly impact the ability of digital devices to capture accurate shade [19]. Therefore, the general recommendation remains to combine digital methods with visual assessment using standardized shade guides for best results in shade selection [20,21].

The absence of quantitative optical reflectance measurements is highlighted in this study. We agree that spectral reflectance curves and chromatic values obtained under standardized conditions, such as D65 illumination, can provide a more complete characterization of the optical behavior of materials. However, we would like to point out that the absence of such data is not unusual in clinical shade-matching studies.

In fact, multiple previous investigations have employed methodologies similar to ours, relying on controlled visual matching and the analysis of CIELAB coordinates or statistical matching indices, without resorting to detailed spectral curves. For example, Gómez-Polo et al. [22] compared visual and instrumental methods using color difference formulae such as CIELAB and CIEDE2000, without including spectral reflectance data. Similarly, Rondón et al. [23] evaluated the agreement between visual methods and standardized photographic analyses, using CIELAB coordinates and color difference formulae, without reporting spectral profiles. Igiel et al. [24] also compared the agreement between visual and instrumental methods, focusing on CIELAB coordinates and color differences, without including reflectance curves. Furthermore, a recent systematic review [25] highlights that many published clinical studies rely on visual observations supported by statistical methods such as the Kappa coefficient, which remains an accepted practice for assessing concordance in tone matching. Consequently, while we recognize that the inclusion of reflectance measures would enrich the analysis, we consider that the methodology used is consistent with a significant proportion of the scientific literature in this area.

Among the study’s limitations regarding sample size determination, we emphasize the exploratory nature of the study and, therefore, a sample of 15 participants is considered adequate. We must always take into account that the objective is to determine methodological feasibility and collect data on color matching, identifying potential trends. Following the ideal sample calculation criteria, the theoretical sample size would be 384 subjects, with a 95% confidence level and a maximum permissible error of 5%. Therefore, future studies should obtain the theoretical sample size to allow for generalized conclusions that will allow for greater rigor.

Another study’s limitation was that the entire study was conducted by dental students, which may present limitations when compared to other studies conducted by more experienced operators.

## 5. Conclusions

This study highlights the limitations of universal composites such as Kuraray Clearfil Majesty ES-2 Universal in color reproduction in posterior teeth, especially in cases with severe dyschromia. Layered composites such as Admira Fusion 5 offer better results in terms of color matching. Furthermore, although digital devices such as the Easyshade spectrophotometer and the Primescan scanner can improve the accuracy of color acquisition, reproducibility remains a challenge, and it is recommended to combine these tools with traditional visual methods to obtain a more aesthetic and clinically acceptable result for the clinician and patient.

Finally, the findings of this study underline the need for further research on the shade matching of biomimetic composites, especially in situations where aesthetic expectations are high. Expanding the sample size and considering factors such as the severity dyschromia and the morphological complexity of the posterior teeth will allow better and clinically relevant results to be obtained. As previous studies have shown, accurate color reproduction in posterior teeth remains a clinical challenge, and although digital methods are advancing, the integration of visual perception remains essential to achieve optimal aesthetic results.

## Figures and Tables

**Table 1:** Descriptive analysis and Cohen’s Kappa Easyshade machine with Admira Fusion.

	N Coincidence	% Coincidence	Kappa	Signification
Pre-Post 1	7	46.7%	0.240	0.064
Pre-Post 2	10	66.7%	0.595	<0.001
Pre-Post 3	10	66.7%	0.654	<0.001

**Table 2:** Descriptive analysis and Cohen’s Kappa of Prime machine with Admira Fusion.

	N Coincidence	% Coincidence	Kappa	Signification
Pre-Post	10	66.7%	0.626	<0.001

**Table 3:** Cohen’s Kappa analysis of the Easyshade machine with Clearfil Majesty.

	Kappa	Signification
Pre-Post 1	0.071	0.261
Pre-Post 2	−0.027	0.481
Pre-Post 3	0.211	0.001

**Table 4:** Cohen’s Prime machine kappa analysis with Clearfil Majesty.

	Kappa	Signification
Pre-Post 1	−0.103	0.155
Pre-Post 2	0.104	0.197
Pre-Post 3	0.272	<0.001

**Table 5:** Descriptive analysis and Kappa on Easyshade machine.

	N Coincidence	% Coincidence	Kappa	Signification
Pre-Post 1	9	30.0%	0.292	<0.001
Pre-Post 2	10	33.3%	0.349	<0.001
Pre-Post 3	14	46.7%	0.469	<0.001
Pre-Post total	33	36.7%		

**Table 6:** Descriptive analysis and Cohen’s Kappa on the Primescan machine.

	N Coincidence	% Coincidence	Kappa	Signification
Pre-Post 1	10	33.3%	0.342	0.064
Pre-Post 2	13	43.3%	0.437	<0.001
Pre-Post 3	15	50.0%	0.508	<0.001
Pre-Post total	38	42.2%		

## Data Availability

The original contributions presented in the study are included in the article, further inquiries can be directed to the corresponding author.
